# Nano-enabled Control of *A. flavus* and *F. proliferatum*: inhibition of fungal growth and mycotoxin biosynthesis by zinc oxide nanoparticles

**DOI:** 10.1038/s41598-026-50553-8

**Published:** 2026-05-06

**Authors:** Elhagag A. Hassan, Ayat H. A. Mohamed Kilany, Ahmed L. E. Mahmoud, Abdulaziz Abu El-fadl

**Affiliations:** 1 Botany and Microbiology Department, Faculty of Science, Assiut University, Assiut, 71516 Egypt; 2 Physics Department, Faculty of Science, Assiut University, Assiut, 71516 Egypt

**Keywords:** *Aspergillus flavus*, Biological control, Fumonisin B_1_, *Fusarium proliferatum*, Mycotoxins, ZnO-NPs, Biochemistry, Biological techniques, Biotechnology, Environmental sciences, Microbiology, Nanoscience and technology, Plant sciences

## Abstract

Recently, zinc oxide nanoparticles (ZnO-NPs) have been proposed as more sustainable substitutes for chemical fungicides; however, their potential in fungicidal and mycotoxin biosynthesis control in food-related pathogens is still under research. In this study, we synthesized and characterized ZnO-NPs with a high degree of crystallinity and antifungal and antimycotoxigenic properties against two significant toxigenic fungi, *Aspergillus flavus* f10 and *Fusarium proliferatum* f30, by using a combination of microwave-assisted combustion. X-ray diffraction (XRD) and FTIR analyses confirmed the formation of a hexagonal wurtzite phase, while TEM imaging revealed rod-like ZnO nanostructures with diameters of approximately 70–90 nm and lengths extending up to approximately 700 nm. ZnO-NPs demonstrated high concentration-dependent fungicidal behavior; 150 ppm decreased the growth of *A. flavus* and *F. proliferatum* by 75 and 97%, respectively whereas bulk zinc sulfate had no effect. SEM revealed severe morphological damage, such as hyphal shrinkage, disturbed sporulation, and outgrowths. ZnO-NPs nearly completely inhibited aflatoxin biosynthesis (reduced AFB_1_ and AFG_2_ by 99-99.9) and partially, but significantly inhibited fumonisin B_1_ synthesis (reduced by approximately 85%), as confirmed by HPLC-analysis. These findings suggest that ZnO-NPs are an effective nanobased intervention to counter the development of fungal contamination and mycotoxin build-up in maize and other food systems. ZnO-NPs offer a promising platform for improving food safety, minimizing post-harvest losses, and promoting sustainable agriculture by simultaneously addressing the proliferation of pathogens and the production of toxins.

## Introduction

Maize (Zea mays L.) is a highly significant cereal crop globally, along with wheat and rice, and contributes almost 60% of the total consumption of carbohydrates and other nutrients worldwide^1^. It has economic and nutritional value not only when it is directly consumed but also when it is commonly used as animal feed, processed foods, and industry products^2^. However, fungal contamination and subsequent mycotoxin buildup pose serious threats to maize productivity and safety^3,4^. Some of these include aflatoxins, which are formed by *Aspergillus flavus*, and fumonisins, which are formed by *Fusarium proliferatum*, and are of special concern^5–7^. These are powerful hepatotoxins, nephrotoxins, and carcinogens associated with liver cancer, immune suppression and reproductive toxicity in animals and humans^8^. Aflatoxin and fumonisin contamination of maize and maize-based products is common in both tropical and temperate areas, causing large post-harvest losses and creating serious health issues in the population^9–11^.

Controlling mycotoxins mainly involves chemical fungicides, decontamination, and physical methods of controlling mycotoxin^12–14^. Chemical treatments can be used to slow the growth of fungal organisms, but this frequently leaves a dangerous residue that is subject to safety and regulatory issues^15,16^. Physical interventions, such as heating or irradiation, can reduce the toxin content; however, they are likely to reduce the nutritional value of grains and are not inexpensive to apply at an industrial level^17,18^. Biological methods based on antagonistic microorganisms or enzymatic degradation have been demonstrated as promising; however, they have been found to be inconsistent in dynamic field conditions^19,20^. These restrictions highlight the pressing necessity for new and sustainable ways to successfully inhibit the development of fungi and the production of toxins, guarantee the safety of food products, and maintain high-quality crops.

Recently nanotechnology has become a revolutionary method in food and agricultural sciences^21,22^. Metal oxide nanoparticles, especially zinc oxide nanoparticles (ZnO-NPs), have received significant interest because of their distinctive physicochemical characteristics, such as nanoscale size, large surface area, and antimicrobial capability^23,24^. ZnO is already a generally recognized as safe GRAS (Generally Recognized as Safe) to the U.S. Food and Drug Administration and has widespread use as a pharmaceutical ingredient, cosmetic, coating, and packaging material^25,26^. There is growing evidence that ZnO-NPs have a broad-spectrum antimicrobial effect against bacteria and fungi with mechanisms that include the production of reactive oxygen species and membrane disruption^23,27,28^. Previous studies have reported that ZnO-NPs exhibit reasonable biocompatibility at low doses^29–31^. Moreover, it has also been proven that green-synthesized ZnO nanoparticles possess antifungal capability against *Fusarium* species, and the decrease in fungal biomass and mycotoxin synthesis of deoxynivalenol and zearalenone has been demonstrated^32^. However, these effects were largely dose-dependent and closely associated with the inhibition of growth.

Nevertheless, important knowledge gaps remain. Few studies have focused on the capacity of ZnO-NPs to prevent the growth of fungi and the production of mycotoxins, which are usually more toxic than fungal biomass itself. In addition, little can be said about their efficiency in dealing with several toxigenic fungi that are frequent co-contaminants of maize. ZnO-NPs have several critical benefits that are not seen in the use of traditional chemical fungicides, which benefits some of the significant limitations of the current control approaches. The fungicides that are being used are mostly chemical in nature, leave longer residues after use, pollute the environment, and even develop fungicides resistance^33^. In comparison, ZnO is known to be GRAS, has high antimicrobial properties at very minimal concentration, and breaks down into forms of zinc that are beneficial in food. Moreover, ZnO-NPs offer double effects by preventing fungal growth and secondary metabolism, whereas the majority of fungicides inhibit the growth of fungi only, without inhibiting toxin synthesis^34^. Their nanoscale dimensions allow them to interact more with fungal surfaces, and their possible addition to food packaging, food coating, and food preservation systems is a significant move toward sustainable, non-residual mycotoxin prevention^35^.

To fill these gaps, the current study compared the antifungal and antimycotoxin properties of ZnO-NPs against *A. flavus* f10 and *F. proliferatum* f30 (two fungi that are important in causing mycotoxin effects in maize). X-ray diffraction, Fourier-transform infrared spectroscopy, and transmission electron microscopy were used to characterize ZnO-NPs synthesized using a microwave-assisted combustion approach. The assessment of their inhibitory action on growing fungi was done using disk diffusion and broth assay, with scanning electron microscopy used to examine the morphological changes. Notably, the effects of ZnO-NPs on the biosynthesis of mycotoxins were measured using high-performance liquid chromatography. By combining structural, morphological, and biochemical studies, this work supports the two-fold properties of ZnO-NPs to inhibit fungal growth and fungal toxin production and underscores the potential of these nanoparticles in global food safety, reducing post-harvest losses, and sustaining agriculture.

## Results

### Characterization of ZnO-NPs

**Fig. 1:**
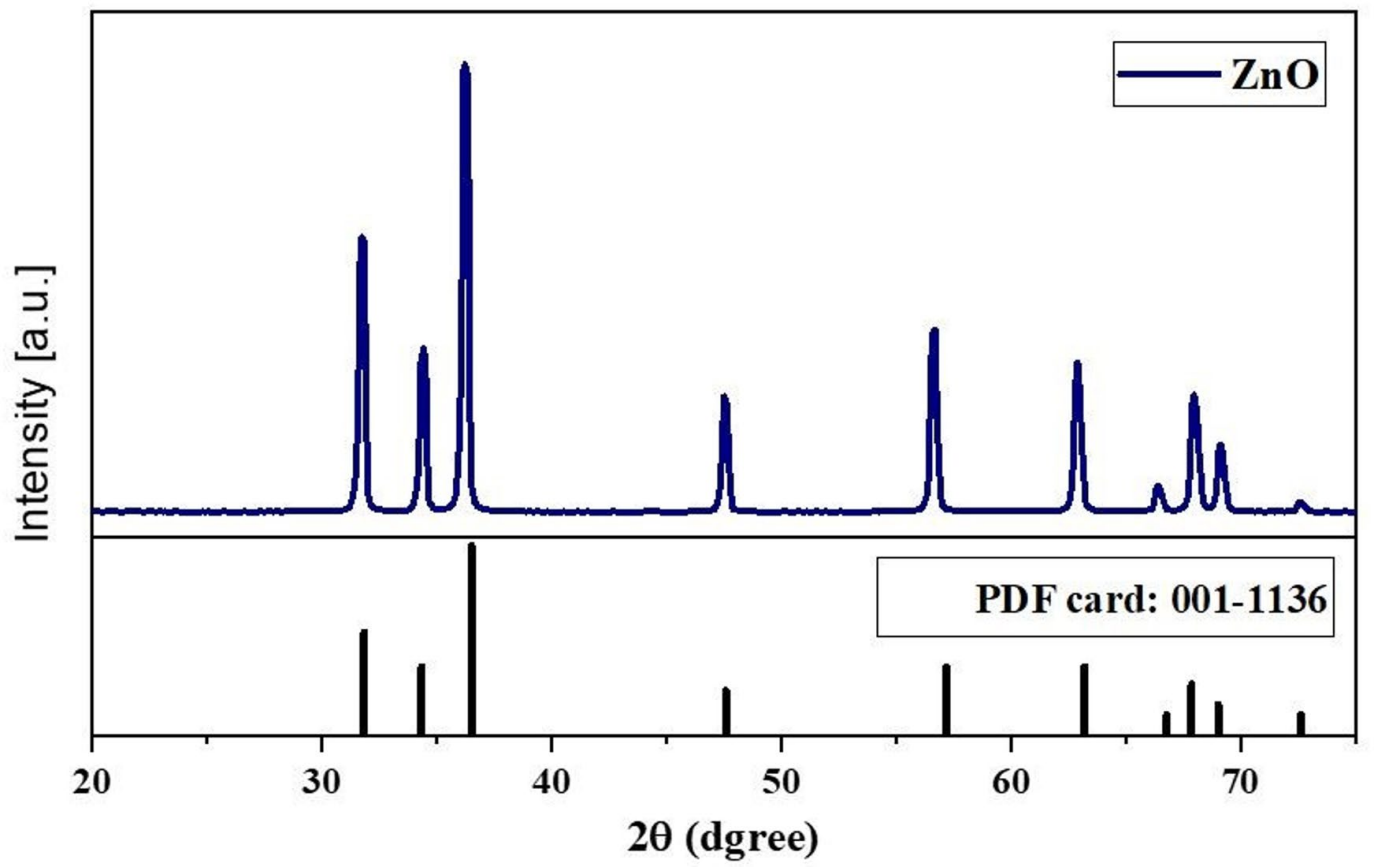
Powder X-ray diffraction patterns of the synthesized ZnO-NPs.

X-ray diffraction (XRD), Fourier-transform infrared spectroscopy (FTIR), and transmission electron microscopy (TEM) were used to characterize the synthesized ZnO nanoparticles. The powder X-ray diffraction data for ZnO nanopowder are shown in Fig. 1, indicating the presence of highly crystalline ZnO nanoparticles with a hexagonal structure (PDF card no: 00-001-1136) and lattice parameters a = 3.242 Å and c = 5.2066 Å. The sharp main peaks at 2θ = 36.52° correspond to the (101) plane, confirming the high crystallinity of the samples.

**Fig. 2:**
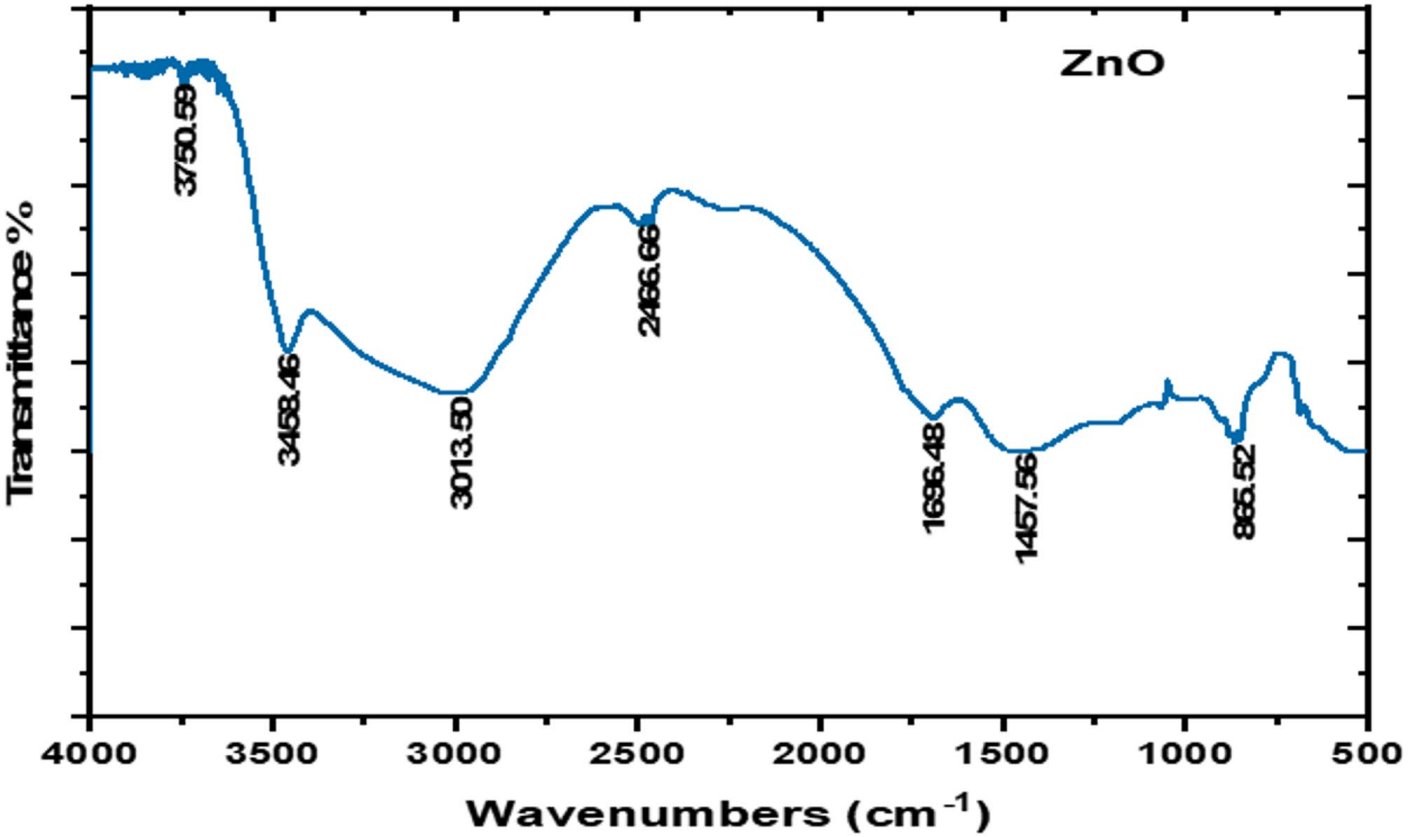
FT-IR spectrum of the synthesized ZnO-NPs.

The FTIR spectra of ZnO-NPs, presented in Fig. 2, revealed significant peaks at 3458 and 502 cm^− 1^. The broad vibrational band at 3458 cm^− 1^ is attributed to the symmetric stretching mode of water molecules, while the peak at 502 cm^− 1^ represents the stretching vibrations of ZnO-NPs and indicates the high purity of the synthesized ZnO nanoparticles.

**Fig. 3:**
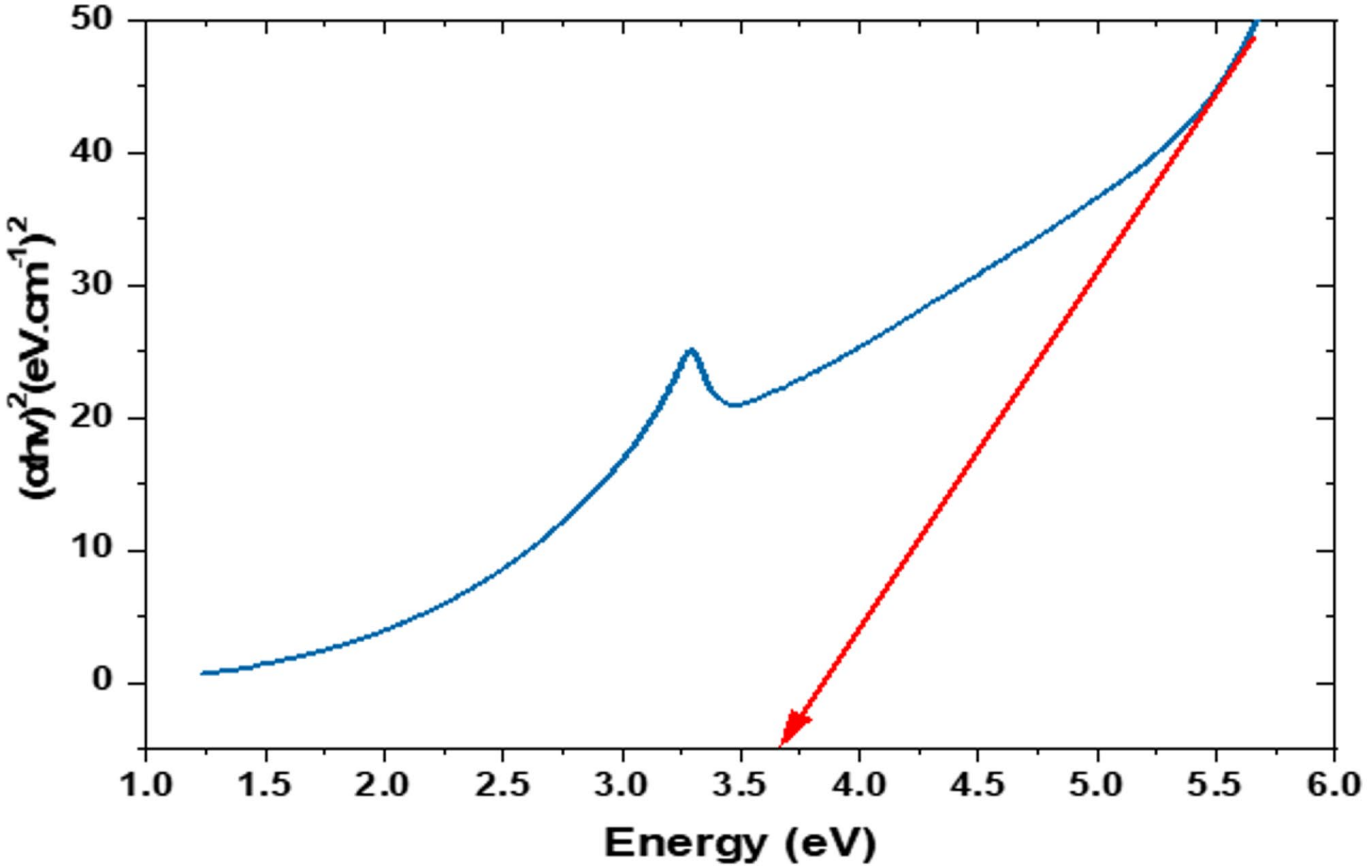
UV-visible spectra of the synthesized ZnO-NPs.

UV-visible spectroscopic analysis was conducted to confirm the synthesis of the ZnO-NPs. The absorption spectrum recorded in the wavelength range of 200–800 nm displayed a characteristic absorption edge at approximately 380 nm, corresponding to a band gap energy of 3.25 eV. The optical band gap, determined from the Tauc plot by extrapolating the linear portion of (αhv)² versus photon energy, was found to be approximately 3.6 eV (Fig. 3).

**Fig. 4:**
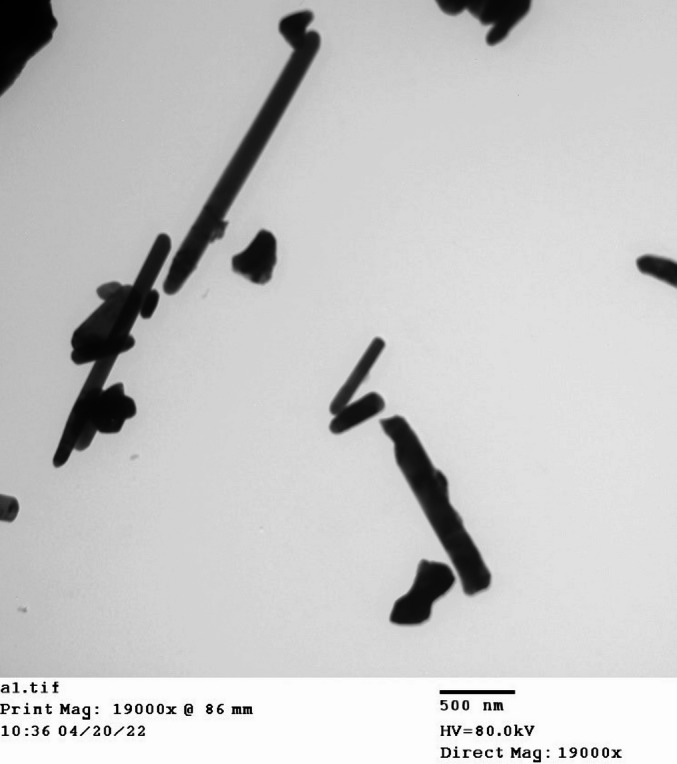
Transmission Electron Microscopy (TEM) graph of synthesized ZnO-NPs sample.

**Fig. 5:**
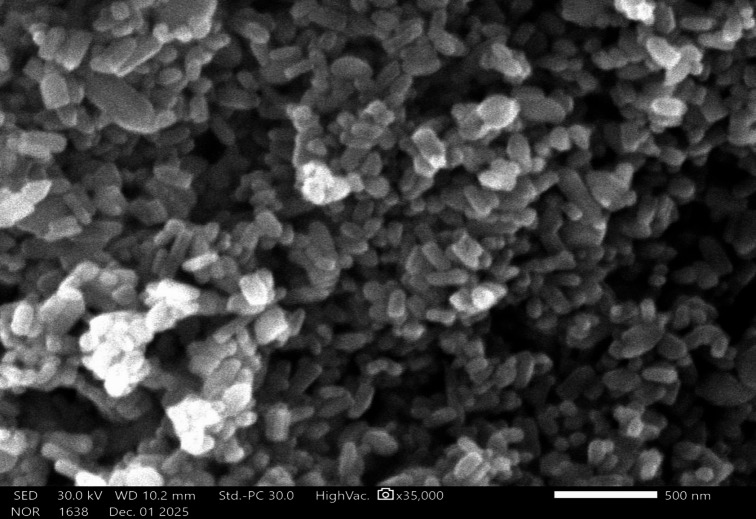
SEM image of ZnO nanoparticles.

Transmission electron microscopy (TEM) analysis was used to determine the size and morphology of the synthesized ZnO-NPs. The TEM micrographs of the ZnO-NPs sample (Fig. 4) revealed the formation of elongated ZnO nanorods with an average of diameter approximately 80 nm and lengths reaching up to 700 nm. In contrast, SEM showed these particles clumped into larger agglomeration in the micron range (Fig. 5). This aggregation behavior is consistent with the hydrodynamic size distribution obtained by DLS analysis, which reflects particle clustering in aqueous suspension rather than the primary particle dimensions observed under TEM.

**Fig. 6:**
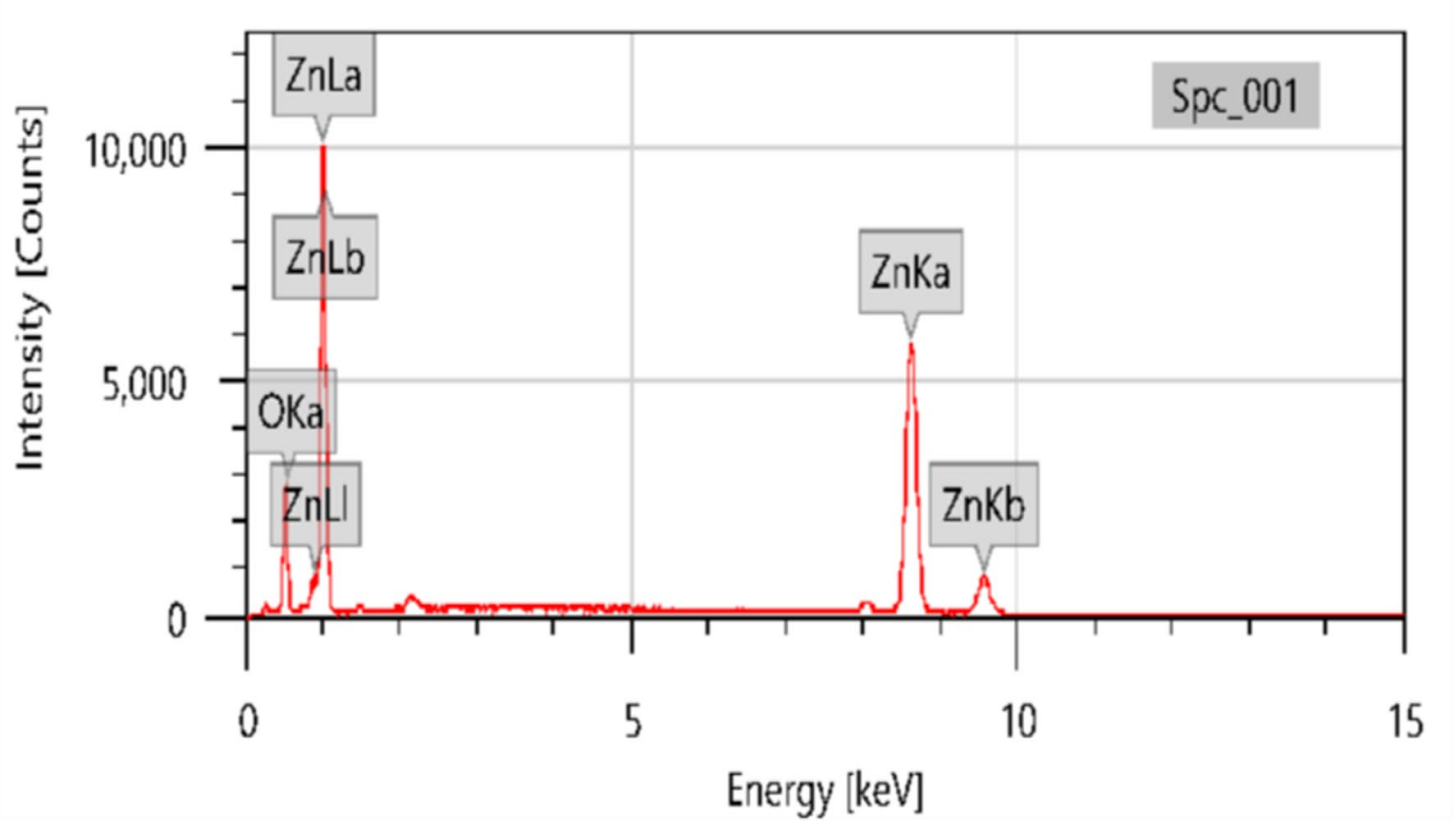
EDX Spectra of Zinc Oxide Nanoparticles.

The purity of the as-prepared nanoscale ZnO particles was further confirmed by energy-dispersive X-ray spectroscopy (EDX). The EDX spectrum of the ZnO nanoparticles is shown in Fig. 6. This spectrum indicates the presence of only Zn and O elements in a 1:1 ratio in the analyzed ZnO sample. The strong peaks shown in the spectra, corresponding to zinc and oxygen, indicate that the synthesized nanoparticles contained only Zn and O elements.

**Fig. 7:**
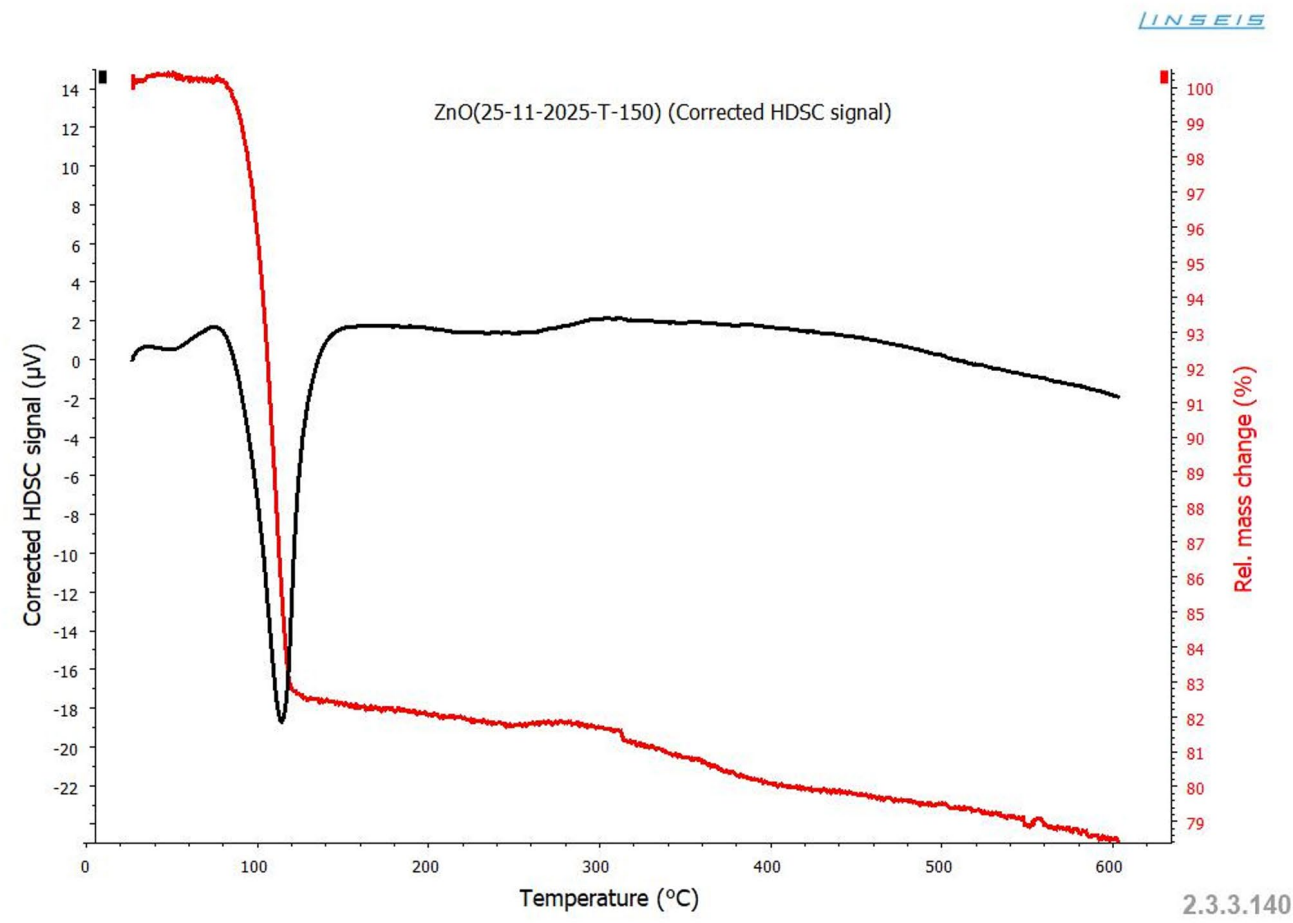
DSC and TGA curves of pure ZnO nanoparticles.

The thermal characteristics were investigated using a Linseis DSC-60 differential scanning calorimeter. The specimens were heated at a scan rate of 10 °C/min over a temperature range of 30–600 °C in a nitrogen atmosphere. The differential scanning calorimetry (DSC) and (TG) are shown in Fig. 7. The data show an endothermic peak with a maximum at *T*_max_ = 119 °C, resulting in a mass loss of 21.5% of the total weight. This loss continues at a slow rate up to 600 °C to yield another 10.5% mass loss, the first peak is assigned to the dehydration of hygroscopic water. The second peak is probably due to the loss of structured water in parallel with a transformation to the crystalline state.

According to the obtained data, in this study, it can be stated that a simple, inexpensive, rapid microwave-assisted combustion method was applied for the synthesis of ZnO nanoparticles.

**Fig. 8:**
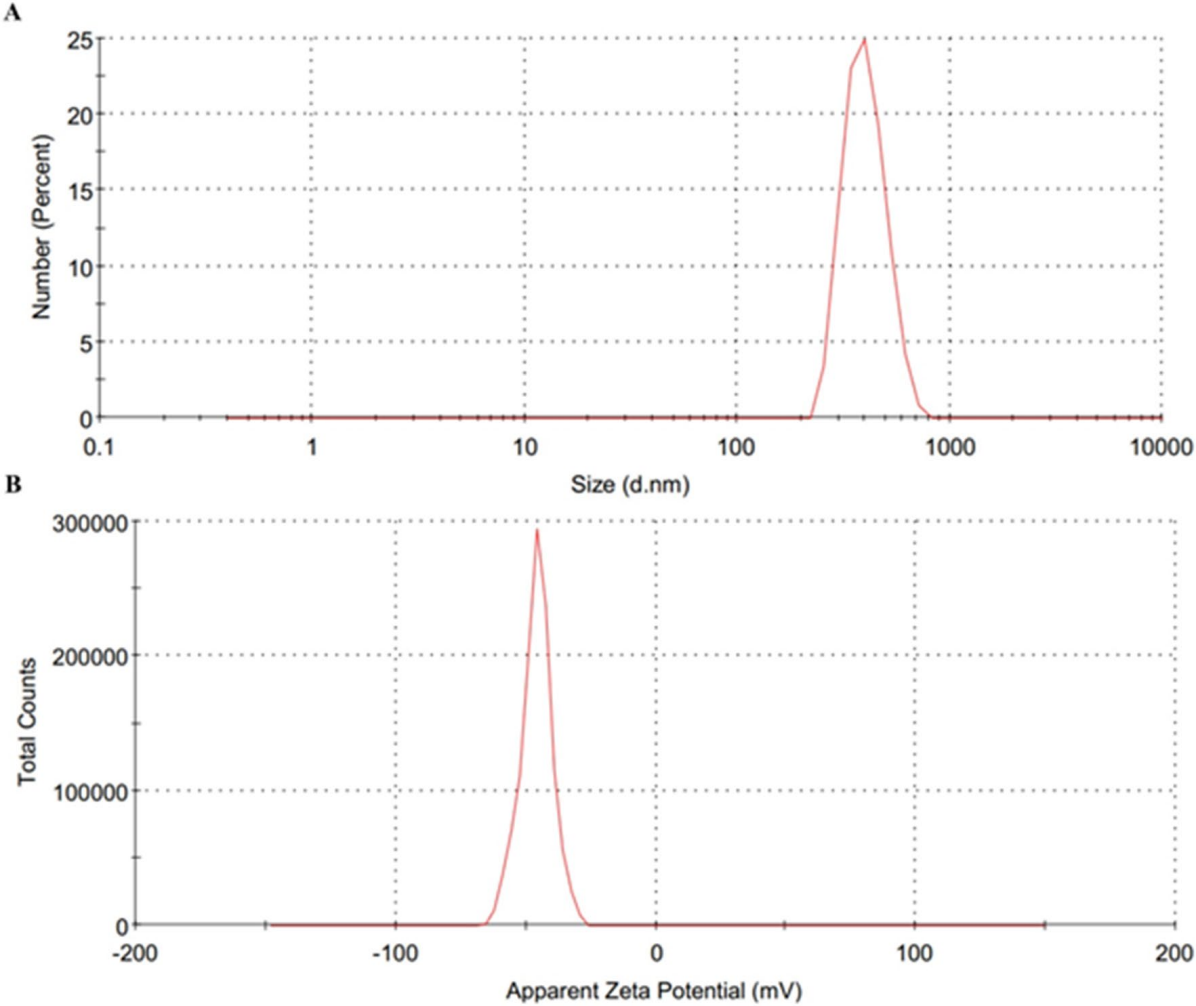
(**A**) Hydrodynamic size distribution of ZnO nanoparticles measured by dynamic light scattering (DLS). (**B**) Zeta potential distribution of ZnO nanoparticles in an aqueous dispersion.

Dynamic light scattering (DLS) analysis was performed to evaluate the hydrodynamic size distribution and surface charge of the synthesized ZnO nanoparticles in an aqueous dispersion. The intensity-weighted size distribution exhibited a primary peak at approximately 404 nm (Fig. 8A). The Z-average hydrodynamic diameter was 917 ± 35.7 nm, with a polydispersity index (PDI) of 0.613 ± 0.042, indicating a broad size distribution owing to aggregation in suspension. The measured zeta potential was − 44.6 ± 1.64 mV (Fig. 8B), suggesting good electrostatic stability of the dispersed particles. The larger hydrodynamic diameter measured by DLS compared to the TEM-observed primary nanorod dimensions (~ 80 nm diameter and ~ 700 nm length) is attributed to particle agglomeration in aqueous medium, as DLS measures the hydrodynamic diameter of particles in suspension rather than their dry-state core size.

### ZnO-NPs antifungal activity

**Fig. 9:**
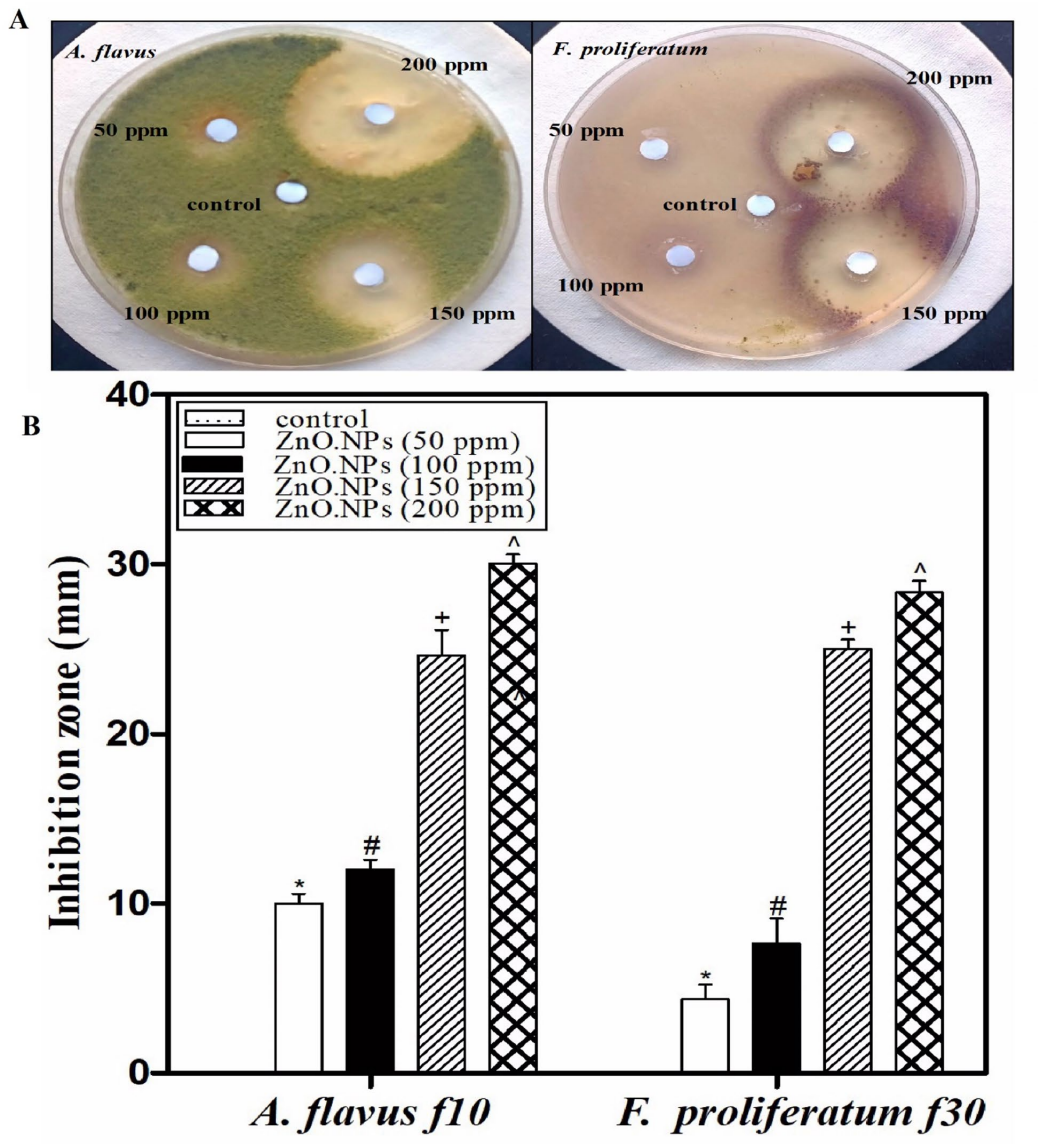
(**A**) Antifungal activity of different concentrations of ZnO-NPs against toxigenic strains (*Aspergillus flavus* f10 and *Fusarium proliferatum* f30) determined using the disk diffusion method. (**B**) Inhibition zone (mm) formed with different concentrations of ZnO-NPs against toxigenic fungi *Aspergillus flavus* f10 and *Fusarium proliferatum* f30.

Zinc oxide nanoparticles (ZnO-NPs) exhibited a distinct dose-dependent antifungal effect against both toxigenic, while bulk zinc sulfate showed no antifungal effect under the same experimental conditions. Disk diffusion tests showed that *Aspergillus flavus* could be inhibited by the presence of ZnO-NPs at concentrations between 50 and 200 ppm. The diameter of the inhibition zone was approximately 10 mm at the concentration of 50 ppm and 30 mm at the concentration of 200 ppm. *F. proliferatum* f30 proved more sensitive, with an inhibition radius of 26 mm at 150 ppm and 29 mm at 250 ppm, which reflected the improved antifungal effect of the nanoparticle with the increase in its concentration, as shown in Fig. 9. Based on these findings, 150 ppm was chosen as the minimum effective concentration (MEC) for future experiments. ZnO-NPs significantly inhibited the production of mycotoxin by both toxigenic fungi at 150 ppm, as they inhibited fungal growth (75% in *A. flavus* and 97% in *F. proliferatum*), indicating strong fungicidal activity and strain-specific sensitivity.

**Fig. 10:**
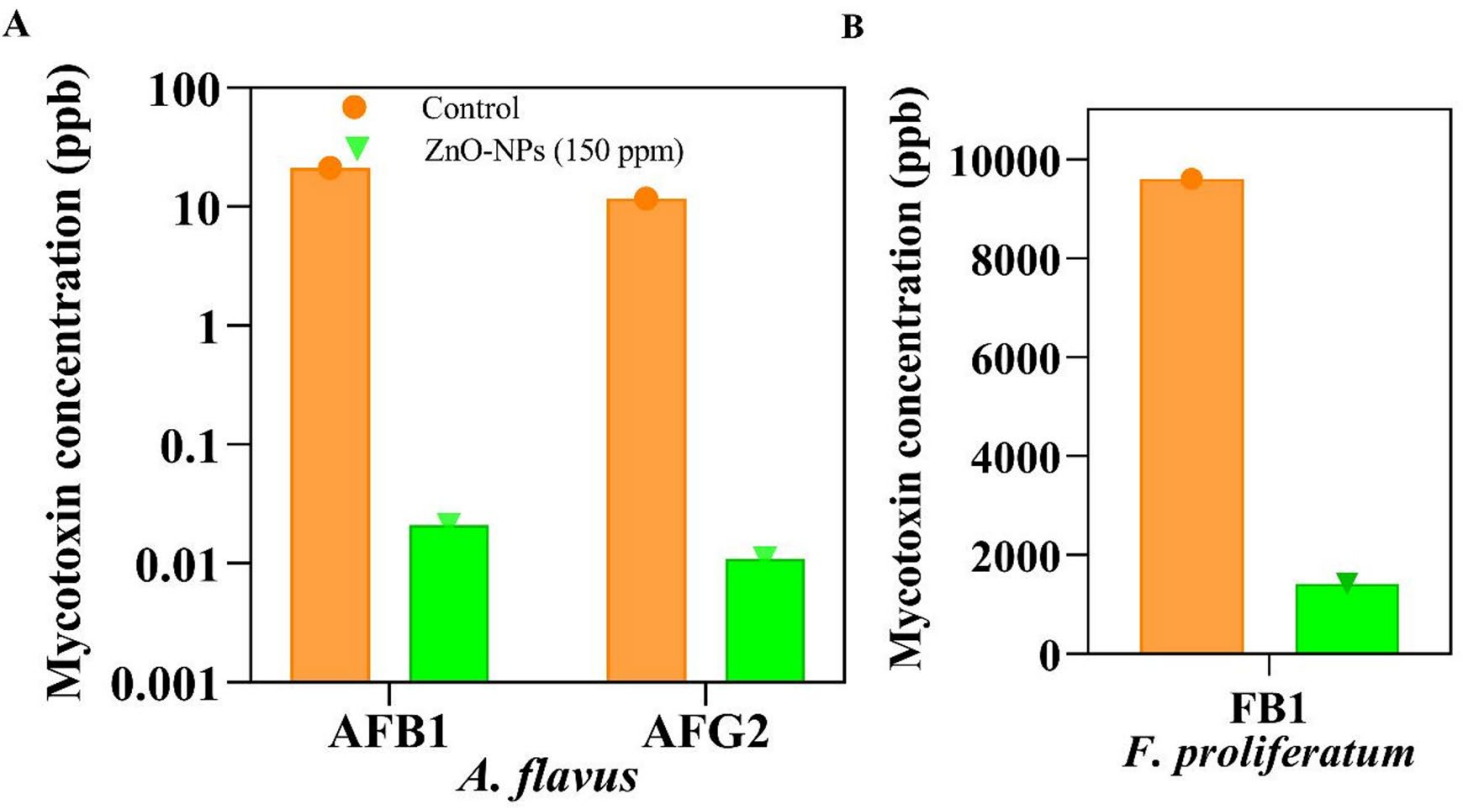
Effect of zinc oxide nanoparticles (ZnO-NPs, 150 ppm) on mycotoxin production by toxigenic fungi. (**A**) HPLC-quantified levels of aflatoxin B₁ (AFB₁) and aflatoxin G₂ (AFG₂) produced by *Aspergillus flavus*. (**B**) Fumonisin B₁ (FB₁) levels produced by *Fusarium proliferatum*. Mycotoxin concentrations are shown for the untreated controls and ZnO-NPs-treated samples.

ZnO-NPs treatment led to a pronounced reduction in the levels of aflatoxin B_1_ (AFB_1_) and aflatoxin G_2_ (AFG_2_) produced by *Aspergillus flavus* compared to untreated controls as illustrated in Fig. 10A. Moreover, exposure to ZnO-NPs markedly decreased the fumonisin B_1_ (FB_1_) production by *Fusarium proliferatum* (Fig. 10B). These observations indicate that ZnO nanoparticles exhibit strong antimycotoxigenic activity against major toxigenic fungi under the tested conditions.

### ZnO-NPs induce morphological changes

**Fig. 11:**
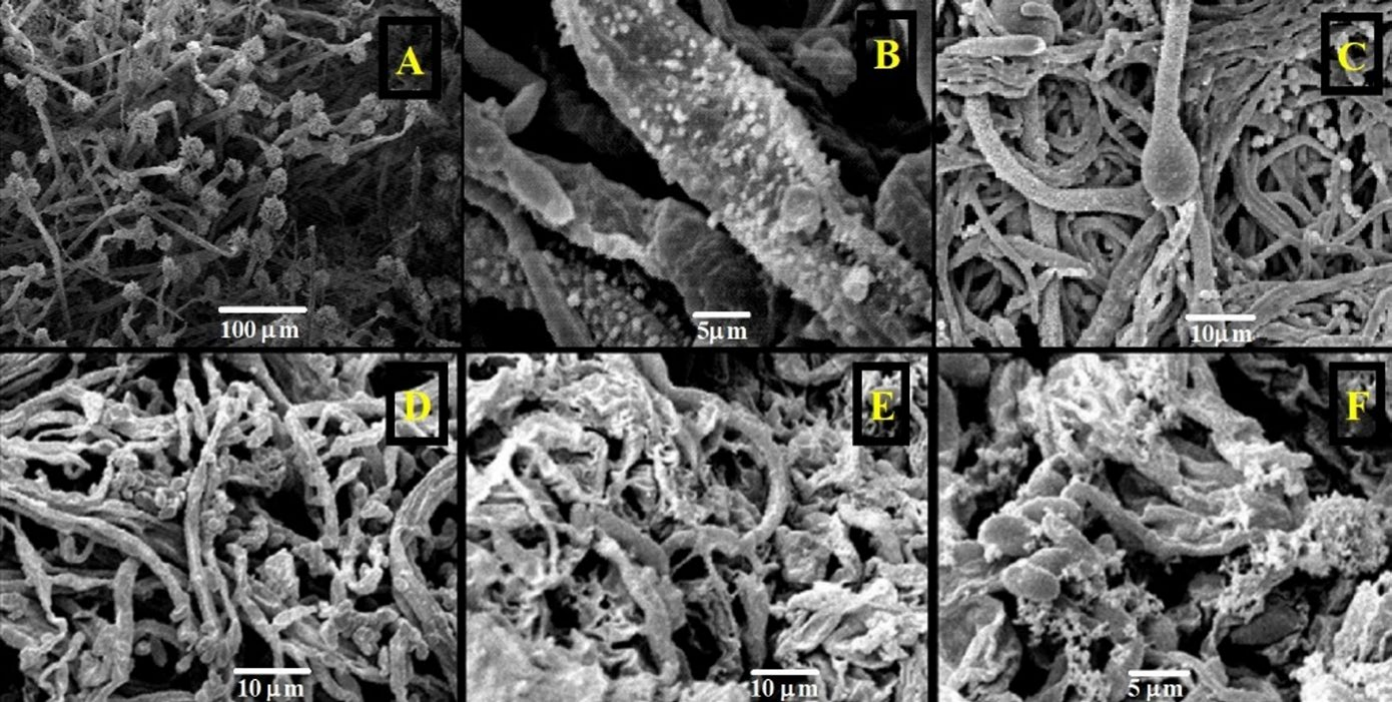
Scanning electron micrographs of tested toxigenic fungi showing (**A**) *A. flavus* f10 growth ˝control˝, (**B**) inhibition of sterigmata and conidia formation of *A. flavus* f10 treated with 150 ppm ZnO-NPs and (**C**) out growth formation and malformations of *A. flavus* f10 mycelia treated with 150 ppm ZnO-NPs, (**D**) *F. proliferatum f30* growth ˝control˝, (**E**) inhibition of sterigmata and conidia formation of *F*. *proliferatum* f30 treated with 150 ppm ZnO-NPs and(F) malformations and shrinkage of *F*. *proliferatum* f30 mycelia treated with 150 ppm ZnO-NPs.

Direct evidence of drastic morphological changes in both fungi after the treatment with ZnO-NPs (150 ppm) was obtained using scanning electron microscopy (SEM) (Fig. 11). Untreated *A. flavus* showed normal hyphae growth with intact conidiogenous structures and abundant conidia production. Conversely, the treated cultures were extensively deformed with collapsed hyphae, shrinkage at the surface, and full inhibition of the development of conidiophore and conidia. Similarly, *F. proliferatum* exhibited healthy filamentous growth in the controls, whereas ZnO-NPs induced mycelial shrinkage, abnormal outgrowths and disturbed spore formation. This mechanistic data on growth inhibition is supported by these structural defects and indicates that ZnO-NPs disrupt fungal cell wall integrity and the ability to sporulate.

### Inhibition of mycotoxin production

HPLC analysis also revealed a significant decrease in toxin biosynthesis. AFB_1_ and AFG_2_ peaks were sharp and severe in untreated cultures, but almost no peaks were present in the samples treated with ZnO-NPs. Similarly, FB_1_ chromatograms indicated that toxin accumulation was significantly lower. These outcomes all testify to the fact that ZnO-NPs suppress the growth of fungi and significantly disrupt the secondary metabolism.

**Fig. 12:**
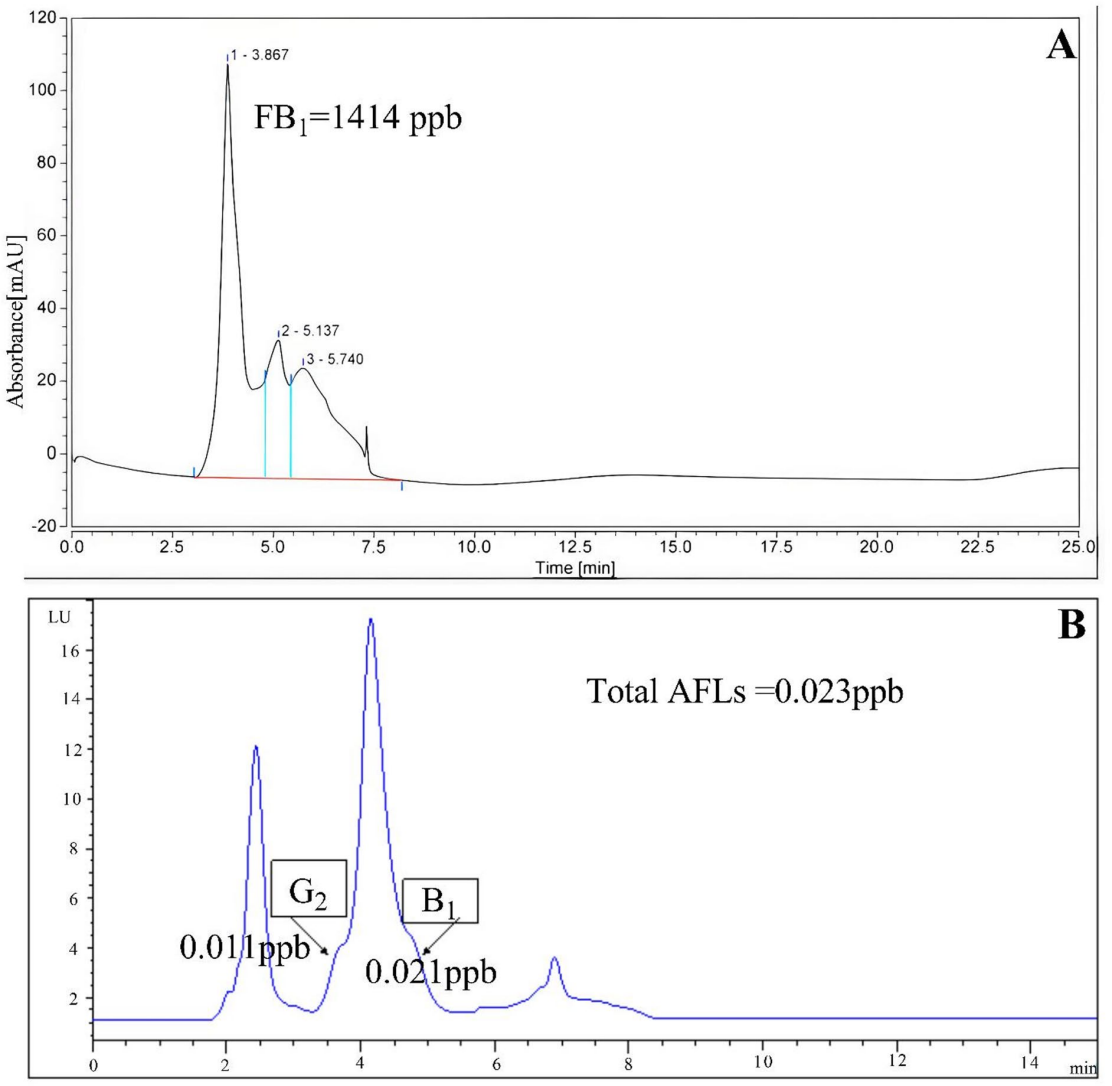
HPLC chromatograms of Fumonisin B_1_ production by *Fusarium proliferatum* f30 (**A**) and aflatoxins production by *Aspergillus flavus* f10 (**B**) after treatment with ZnO-NPs (150 ppm).

ZnO-NPs of 150 ppm significantly suppressed mycotoxins production. In the untreated controls, *A. flavus* generated 21.2 ppb of AFB_1_ and 13.6 ppb of AFG_2_, but in the case of ZnO-NPs treatment, 21.2 ppb and 13.6 ppb were inhibited by 99.9% and 99%, respectively, indicating that ZnO-NPs treatment practically inhibited *A. flavus*. In the control cultures, *F. proliferatum* synthesized 9600 ppb FB_1_, which decreased to 1414 ppb upon exposure to ZnO-NPs, representing an 85% decrease. In this way, aflatoxin was practically lost, and fumonisin B_1_ was partially and significantly decreased. One-way ANOVA and post hoc test (Tukey) showed these results to be statistically significant (*p* < 0.05). HPLC chromatograms, as shown in Fig. 12, also verified that the peaks of aflatoxins and the signal intensities of fumonisin were almost completely disinhibited and dimmed in the samples subjected to ZnO-NPs treatment, respectively. Notably, the dual nature of fungus suppression and toxin biosynthesis warrants the possibility of ZnO-NPs to improve the risks of food contamination compared to other antifungal approaches that fail to recognize toxin biosynthesis.

## Discussion

The current study demonstrates the antifungal and antimycotoxigenic effects of zinc oxide nanoparticles (ZnO-NPs) against two predominant foodborne pathogens, *Aspergillus flavus* and *Fusarium proliferatum*. ZnO-NPs inhibited fungal growth by 75 and 97% at 150 ppm and nearly entirely blocked aflatoxin production and significantly lowered the level of fumonisin. These results imply the potential of ZnO-NPs as an innovative dual-action approach to reduce fungal contamination and mycotoxin biosynthesis in maize-based systems.

The antifungal activity of the study aligns with previous reports that note that ZnO-NPs can subdue pathogenic fungi including; *A. niger*, *A. ochraceus*, and *Rhizopus* spp. by disturbing cell walls and by preventing sporulation^36^. Nevertheless, the degree of mycotoxin inhibition obtained in this study (99% aflatoxins and 85% fumonisin B_1_) is greater than that of most antifungal agents used in the past (chemical fungicides and adsorbents, including hydrated sodium calcium aluminosilicate (HSCAS)^37–39^. ZnO-NPs provide more comprehensive protection against fungal growth and secondary metabolism, unlike adsorbents that bind toxins only after the production process. This twofold operation makes ZnO-NPs a better option than current approaches that do not always manage to reduce the proliferation of pathogens and, at the same time, toxin production.

The strong morphological distortions caused by SEM, such as hyphal collapse, abnormal outgrowths, and inhibited sporulation, indicate that ZnO-NPs affect fungal cell wall integrity and disrupt developmental processes. These effects can be attributed to several mechanisms. First, ZnO-NPs produce reactive oxygen species (ROS), including hydrogen peroxide, which cause oxidative stress and damage cellular components^40^. Second, Zn^2+^ ions that leak out of the nanoparticles can interfere with intracellular signaling and disrupt membrane permeability^41^. Third, direct physical contacts between nanoparticles and the fungal surfaces could inhibit membrane function and prevent nutrient uptake^42^. Collectively, these processes are likely to play a part in the inhibited growth of fungi and the synthesis of toxins.

Previous studies on ZnO nanoparticles mostly indicate a concomitant reduction in fungal growth and mycotoxin production, which implies that toxin inhibition is more of a by-product of biomass inhibition^32^. Conversely, the current research shows that inhibition of aflatoxin and fumonisin biosynthesis is particularly disproportionately inhibited in comparison with fungal growth, suggesting a specific pattern of secondary metabolic pathway inhibition as opposed to a fungicidal effect. Furthermore, whereas previous research focused on *Fusarium graminearum*, and food-borne phytopathogenic fungi and did not assess mycotoxin biosynthesis^43^, our research expands these resources to major foodborne toxigenic fungi (*Aspergillus flavus* and *Fusarium proliferatum*), which are directly relevant to food safety and human health. The results of the antifungal and antimycotoxigenic effects of zinc oxide nanoparticles reported in this study are consistent with and even greater than those reported previously. For example, previous researchers who employed ZnO-NPs or other formulations of nanometals listed 40–70% death at toxin concentrations. However, in the present study, 99% of aflatoxins and 85% of fumonisin B_1_ were reduced at only 150 ppm. This degree of inhibition indicates that ZnO-NPs influence growth and secondary metabolism through a simultaneous effect. The extreme morphological abnormalities observed under SEM, such as collapsed hyphae, disrupted conidiophores, and abnormal outgrowths, contribute to the use of a mechanism based on membrane damage and oxidative stress. Such structural defects would most probably perturb intracellular signaling pathways that control toxin biosynthesis. In addition, the release of Zn^2+^ ions and the production of ROS can disrupt the activity of the polyketide synthase complex, thus preventing aflatoxin and fumonisin synthesis pathways. The status of nanoparticle-mediated inhibition of toxin biosynthesis is a major breakthrough in post-harvest food safety measures compared to conventional fungicides, which mostly act through growth inhibitory effects.

This study has shown that ZnO-NPs have a dual mode of action because of the relationship between the inhibition of fungal biomass and the inhibition of mycotoxin biosynthesis. The 75% and 97% fungal growth inhibition in *A. flavus* and *F. proliferatum*, respectively, was disproportionate to the decrease in biomass, although aflatoxins (> 99%) and fumonisin B_1_ (approximately 85) were inhibited correspondingly. This indicates that ZnO-NPs do not merely suppress the fungal growth. The gross structural deformities observed in SEM, such as hyphal collapse and secondary metabolism impairment, are indicative of ZnO-NPs disruption of the physiological processes that occur during secondary metabolism. In addition, oxidative stress caused by the formation of ROS and the release of Zn^2+^ ions is likely to disrupt transcriptional regulation of toxin biosynthetic clusters. Thus, the biosynthesis of toxins seems to be a more critical response to the stresses of nanoparticles than primary biomass growth, which is why the antimycotoxigenic effect is also strong in cases when fungal growth does not cease.

In addition to antifungal activities, ZnO-NPs have viable benefits for food and agricultural purposes and can play a key role in nano-enabled effective and sustainable agricultural implementations^44^.

To ascertain whether ZnO nanoparticles might inhibit secondary metabolism at the lowest practical dose, mycotoxin formation was measured at the lowest concentration showing strong inhibition. Although the current research was based on the antifungal and antimycotoxigenic activity of ZnO nanoparticles, biocompatibility should be considered for the potential application of nanoparticles. Previous research has revealed that ZnO-NPs have moderate biocompatibility at low doses, and cytotoxicity is usually dose-dependent and depends on the size of the particle, surface characteristics, and route of exposure. ZnO-NPs are frequently suggested for agricultural and food safety applications, likely because they are used indirectly, for example, as a surface treatment, packaging, or controlled-release system, and they may not expose individuals directly to them^29,30^. However, full toxicological assessment under real exposure conditions is a significant direction for future research. Zinc oxide is considered safe and is classified as GRAS by the U.S. FDA and is already used in food wrapping, surface finishes, and pharmaceutical preparations^45^. However, the toxicity and environmental fate of nanoparticles should be considered. Although some studies have found a low level of cytotoxicity with ZnO-NPs at low doses, long term exposure to them can cause a buildup of zinc in tissues, which can create safety-related concerns that need to be overcome by conducting rigorous toxicological studies. ZnO-NPs also showed high-antimycotoxigenic properties in the low concentration used in this study,150 ppm; therefore, it is possible to suggest that effective toxin suppression can be observed at lower doses compared to those related to cytotoxicity.

The experiment showed that there was a difference in their responses to the toxin classes, with near-complete inhibition (> 99%) and significant yet moderate inhibition (almost 85%) in the production of FB_1_. This difference indicates that aflatoxin biosynthesis is more prone to oxidative and structural stress caused by ZnO-NPs than fumonisin biosynthesis, which might require more nanoparticles or longer exposure duration to be fully suppressed. Notably, the concentrations used in this study (not more than 150 ppm) are not that high, and this might reduce the possible risks when properly adjusted to practical scenarios.

Although no direct measurement was performed on the levels of gene expression in this study, the fact that AFB_1_, AFG_2_ and FB_1_ production was strongly suppressed by ZnO-NPs indicates that ZnO-NPs perturbs the transcriptional regulation of mycotoxin-biosynthetic pathways. Past research has shown that oxidative stress and ROS formed by ZnO-NPs are capable of downregulating major regulatory genes (aflR and aflS) that control the expression of over 25 genes that are in the aflatoxin cluster^46,47^. Likewise, there are transcription factors that regulate fumonisin biosynthesis, including FUM21, polyketide synthase FUM1 and aminotransferase FUM8^48,49^. These genes are highly susceptible to redox imbalance and membrane stress. The drastic morphological damage observed in SEM (hyphal collapse, disrupted sporulation and abnormal branching) indicates that membrane integrity is impaired and can affect signal transduction pathways, triggering secondary metabolism. Zn^2+^ leakage from nanoparticles can also disrupt intracellular regulatory pathways in toxin biosynthesis. Thus, almost complete inhibition of aflatoxins and the substantial decrease of fumonisin B_1_ were achieved in the current study, which can be explained by a mechanistic model, according to which ZnO-NPs disorient both the growth and transcriptional machinery needed by fungi to produce secondary metabolites.

Nanoparticle strategies, specifically ZnO-NPs, offer several advantages over conventional chemical fungicides. To begin with, ZnO-NPs have fungicidal and antimycotoxigenic effects, and the majority of fungicides inhibit the biomass of fungi but do not interfere with toxin production^50^. Second, nanoparticles are less prone to cause resistance development owing to the presence of multiple cellular targets by their modes of action, which include ROS generation, membrane disruption, ion release^51^. Third, ZnO-NPs have fewer chemical residue issues and less environmental persistence, making them a safer substitute for food/feed protection^52^. Finally, ZnO-NPs can also be used in smart packaging, nano-coatings, or surface disinfectants, allowing continuous release with immobilized antimicrobial activity by such products, which is not possible with other fungicides^53^.

Although ZnO-NPs can be used as promising antifungal and antimycotoxigenic agent, the potential cytotoxic and ecotoxic effects of ZnO-NPs should be considered. These risks can be addressed using several practical strategies. First, minimal effective concentrations must be used. The concentration of 150 ppm was selected as the lowest concentration showing strong inhibition, and it was far below the concentrations which are linked with toxicological issues in the human or animal system. Second, the migration of nanoparticles can be inhibited by immobilizing ZnO-NPs in polymeric packaging films, edible coatings, or nanocomposites; thus, immobilization of the nanoparticles and preventing direct exposure to the nanoparticles can still confer antimicrobial activity. Additionally, uncontrolled dissolution of Zn^2+^ ions is a significant source of cytotoxicity that can be decreased by surface modification of ZnO-NPs. Furthermore, biopolymers or lipid delivery can be used to regulate the release rates and enhance the environmental friendliness. Finally, any large-scale application must be accompanied by comprehensive environmental risk evaluation, which involves studying soil accumulation and the impact of the application on non-target organisms. A combination of these strategies offers ways in which the advantages of ZnO-NPs can be tapped without causing harm to human health and safety or the environment.

Despite the benefits of ZnO-NPs, their use in agriculture and food production has profound ecological and toxicological effect. Nanoparticles may be permanent in soil and water and may alter the composition of microbial community or interfere with nutrient cycling. The accumulation of ZnO-NPs in soil has the potential to affect earthworms, non-pathogenic fungi, and nitrogen-fixing bacteria, leading to oxidative stress or membrane damage. Moreover, it influences the possibilities of trophic transfer, where nanoparticles bypass the food chain and are deposited in more complex food chain stages. Although ZnO is considered GRAS and can be considered safe in small amounts, excessive release of Zn^2+^ ions can cause cytotoxicity to mammalian cells, or phytotoxic effects on sensitive crops. Nano-specific risk assessment, including assessing of particle size, dissolution rates, route of exposure, and long-term ecological effects, is increasingly mandated by regulatory bodies. The concentrations applied in this study were relatively low (150 ppm), but they did not exceed the reported safe concentrations, and further ecotoxicological research is needed to ensure responsible and sustainable consumption^54^.

The antifungal activity of ZnO-NPs may change significantly under diverse environmental parameters, which has significant implications for the practical use of these products. One of the key determinants is humidity; increased humidity promotes the interaction between nanoparticles and fungus and indicates Zn^2+^ ion dissolution, causing the augmentation of oxidative stress and antifungal activity, but the opposite is also true. In other words, elevated pH suppressed the nanoparticle reactivity. Temperature determines the properties of nanoparticles and the physiology of fungi. High temperatures tend to increase the amounts of ROS formed by ZnO-NPs and could also promote their inhibitory ability, as well as stress fungal cells^55^. Conversely, low temperatures can reduce the reaction ability of nanoparticles, thereby inhibiting fungal metabolism. These ecological necessities imply that the ZnO-NPs usage is possibly most practical in circumstances that resemble warm and damp storing settings, in which mycotoxin-generating fungi thrive the most.

In the future, the incorporation of ZnO-NPs into food packaging, grain storage, and agricultural surface has the potential to minimize post-harvest losses and increase food security. Future studies should focus on up-scaling these results to the field, determining the stability of nanoparticles in complex food matrices, as well as the safety of nanoparticles for consumers and the environment in the long term. In addition, the study of synergistic activity of ZnO-NPs with other natural antifungal compounds, including essential oils or probiotic microorganisms, can serve to improve the performance of nanoparticles and decrease their doses.

Overall, this study provides strong experimental evidence that ZnO-NPs can simultaneously inhibit the growth of pathogenic fungi and aflatoxin and fumonisin B_1_ biosynthesis. By integrating structural characterization, antifungal tests, morphological studies, and toxin evaluation, this study provides an overall platform on explaining ZnO-NPs as dual-effort antifungal agents. These findings underscore their potentials as a safe, sustainable, and innovative solution to the ongoing problem of fungal contamination and accumulation of mycotoxins in maize, and related food systems.

This research paper adds to the growing research chain of nano-enabled food safety knowledge, as it has shown that ZnO-NPs offer a dual-response approach that can suppress fungal growth as well as mycotoxin production. Compared to traditional fungicides or detoxification treatments of post-harvest that consider only one part of the contamination, ZnO-NPs disrupt the growth and secondary metabolism simultaneously. Such a combination mechanism makes ZnO-NPs an ideal platform upon which it could be brought into future use in active antimicrobial package, nano-coating of grain storage, seed protection treatments, and surface-sanitization strategies. The high potency of ZnO-NPs at low concentrations justifies the possibility of integrating ZnO-NPs into commercial and scaled non-thermal and non-residue mycotoxin prevention methods. Hence, our results represent a significant step towards the development of viable, sustainable nano-enabled solutions in mitigating foodborne-related risks and bolstering post-harvest safety.

The repression of toxin biosynthesis compared to biomass reduction is disproportionate, indicating that ZnO-NPs activate a specific set of regulatory responses required by secondary metabolism, which can be viewed as a mechanistic explanation for their high antimycotoxigenic activity.

## Conclusion

It has been shown that zinc oxide nanoparticles (ZnO-NPs) are highly effective in the simultaneous inhibition of toxigenic fungi (*Aspergillus flavus* and *Fusarium proliferatum*) as well as their toxins (aflatoxins and fumonisin B_1_). ZnO-NPs caused severe structural damage to fungal hyphae, inhibited sporulation, and inhibited toxin production up to 99% at relatively low concentrations (150 ppm). These results position ZnO-NPs as dual-activity antifungal agents that can be used to address proliferation and secondary metabolism of pathogens.

By combining the characterization of nanoparticles, antifungal studies, microscopic analysis and quantification of toxins, this research provides a complete outline of the application of ZnO-NPs in agronomy and food safety. ZnO-NPs, owing to their high antimycotoxinogenicity, have the potential to be used as sustainable alternatives to traditional fungicides and adsorbents, with the added benefits of reducing post-harvest losses and preserving crop quality. To achieve safe deployment, mitigation measures, including dose reduction, nanoparticle packaging, and controlled release formulations should be used in future applications of ZnO-NPs to minimize cytotoxicity or environmental exposure. Under these precautionary measures, ZnO-NPs can be utilized in a responsible manner as a nano-enabled system to manage fungal contamination and mycotoxin accumulation issues.

Additional studies should be conducted to confirm the effectiveness of ZnO-NPs in field and storage environment, their stability in complex food conditions, and long-term biosafety in humans, and the environment. ZnO-NPs may be utilized as a new nano-enabled solution to the issue of fungal contamination and mycotoxin accumulation in food and possibly used as barrier against fungal infections in food packaging, grain storage system and crop protection strategy with cautious optimization and regulatory evaluation.

This study contributes to the overall concept of nano-enabled food safety by demonstrating the simultaneous antifungal and antimycotoxin effects of the system. ZnO-NPs can be considered an attractive platform for designing active packaging systems, nano-coatings, and post-harvest protection solutions aimed at decreasing fungal infections and fungal toxins. The results inform the future on the way forward of a translational study on the application of a safe and sustainable nanotechnology-based solution to food and agricultural systems.

## Materials and methods

### Synthesis of ZnO-nanoparticles

Pure ZnO nanoparticles are synthesized using the microwave combustion method. In this process, stoichiometric amounts of zinc nitrate and urea were dissolved in deionized water and poured into a quartz container can be mixed well by magnetic stirring for 1/2 h, which makes them almost as homogeneous mixtures, which was placed in a domestic microwave oven (Olympic electric (KOR-6Q1B) Microwave 20 L, 800 W), input range 210–230 V-ac 50 Hz, operating for 5 min. Initially, the solution boiled and underwent dehydration, followed by decomposition with the evolution of a large amount of gases with white fumes coming out of the exhaust opening provided on the top of the micro oven. After the solution reached the point of spontaneous combustion, it begun to burn and released a large amount of heat, vaporizing all the solution instantly and becoming a foamy white solid powder. After synthesis, the precipitate containing ZnO-NPs was washed repeatedly with distilled water and ethanol to remove unreacted precursors and impurities such as salts or hydroxides. Centrifugation is then used to separate the solid NPs from the liquid, and this washing and centrifugation process may be repeated. Finally, the purified ZnO-NPs are dried, often in a hot air oven at temperatures ranging from 80 to 100 ℃, to remove residual solvents^56^.

### Characterization of ZnO-nanoparticles

Phase analysis was conducted using room-temperature powder X-ray diffraction (Model Philips PW 1710) with CuKα radiation (λ = 1.5405 Å) at an operating voltage of 40 kV and a current of 30 mA. The scanning rate was maintained at 0.06° per minute over the 10º-75º 2θ range. Fourier-transform infrared spectroscopy (FTIR) was performed using the KBr method on a Nicolet spectrophotometer (model 6700) in the wavenumber range of 500–4000 cm^− 1^. The fine powder was examined using a JEOL TEM (Model 100 CX II; Tokyo, Japan) at the Electron Microscopy Unit, Assiut University, Egypt, after dissolution in ethanol^56,57^. A 5 mg sample was dispersed in 50 mL of deionized water (100 mg/L), and bath-sonicated for 10 min immediately prior to measurement. The prepared particles were analyzed for their particle size and size distribution in terms of the average volume diameters and polydispersity index by photon correlation spectroscopy using particle size analyzer Dynamic Light Scattering (DLS) (Zetasizer Nano ZN, Malvern Panalytical Ltd, United Kingdom) at a fixed angle of 173° at 25 °C. The samples were analyzed in triplicates. The same equipment was used to determine the zeta potential.

### Toxigenic fungi and their mycotoxins

*Aspergillus flavus* strain f10 and *Fusarium proliferatum* strain f30 were isolated from yellow corn and cornflakes, respectively^58^. They were cultured on potato dextrose agar (PDA) at 28 °C for a week before use, following the protocol by^59^. Molecular identification based on 18 S rRNA was conducted by the mycological center at Assiut University, Egypt. The designations f10 and f30 refer to laboratory isolate codes used for strain identification. The obtained sequences of toxigenic fungal strains *Aspergillus flavus* f10 *and Fusarium proliferatum* f30 were deposited in the GenBank database under accession numbers OQ087136 and OQ087105, respectively.

To evaluate the maximum inhibition of toxin biosynthesis under growth-inhibitory conditions, mycotoxin quantification was conducted at the lowest concentration showing strong inhibition (150 ppm). Quantitative analysis of mycotoxins was performed using high-performance liquid chromatography (HPLC). Aflatoxins were detected using a fluorescence detector at an excitation wavelength of 365 nm and an emission wavelength of 455 nm, using a mobile phase of Water: Acetonitrile: Methanol (55:30:15, v/v/v). Fumonisin B_1_ was detected with an excitation wavelength of 335 nm and an emission wavelength of 440 nm using a mobile phase of Water: Acetonitrile: Acetic acid (59:4:1, v/v/v) and Acetonitrile: Acetic acid (99:1, v/v) for solvents A and B, respectively. The crude extract was analyzed using an HPLC apparatus (Agilent Technologies 1200 Series, G1321A FLD with column Zorbax Eclipse Plus C18) at the Analytical Chemistry Unit at Assiut University.

### Determination of fungicidal activity of ZnO-NPs on the growth of toxigenic fungal strains

The antifungal activity of ZnO-NPs against toxigenic strains of *A. flavus* f10 and *F. proliferatum* f30 was evaluated using the disk diffusion method with sterilized paper disks^60^. Spore and fungal spore suspensions of the tested toxigenic fungi (200 µl containing approximately 10^7^ spores per ml) were aseptically spread onto PDA plates. Disks (5 mm) containing freshly prepared ZnO-NPs at different concentrations (50, 100, 150, and 200 ppm) were prepared, with zinc sulfate (bulk material) used as the control. Five disks were placed in each petri dish, and the plates were incubated at 26 ± 2 °C for five days. The inhibition zones (mm) were measured to assess the effects of ZnO-NPs treatment^61^. The antifungal activity of ZnO nanoparticles was evaluated using the disk diffusion assay as a preliminary screening method. However, because nanoparticles exhibit limited diffusion through agar matrices, inhibition zone diameters were interpreted comparatively rather than as absolute minimum inhibitory concentration (MIC) values. Therefore, the term MIC is not appropriate in this context. Instead, 150 ppm was selected as the minimum effective concentration (MEC), representing the lowest concentration that produced pronounced growth suppression in the tested fungal strains. A true MIC, defined as the lowest concentration resulting in complete inhibition of visible growth under standardized broth microdilution conditions, was not determined in this study. The selection of 150 ppm allowed assessment of antifungal efficacy while still permitting sufficient fungal biomass for subsequent evaluation of mycotoxin production under nanoparticle-induced stress. The assays were performed in triplicates.

### Impact of ZnO-NPs on the toxigenic fungal growth

The fungal growth margins near the clear zone at 150 ppm, which was the lowest concentration showing strong inhibition, were examined using scanning electron microscope (SEM) to observe the morphological changes in mycotoxigenic strains induced by ZnO-NPs. A 10 mm disc of the fungal growth margin was cut and fixed in 5% cold buffered glutaraldehyde for 2 days. Subsequently, the samples were post-fixed in 1% osmium tetroxide for two hours, followed by three rinses (30 min each) in sodium cacodylate buffer. The samples were then dehydrated using an ascending ethanol gradient (30%, 50%, 70%, and 90%) for two hours, 100% ethanol for two days, and amylacetate for two days. The samples were dried using liquid carbon dioxide in a critical point drainer, attached to metallic blocks with silver paint, and coated with a 15 nm layer of gold using a gold sputter device. Finally, the samples were analyzed and imaged at 15 kV using a JEOL JSM 5300 Lv scanning electron microscope^62^.

### The impact of ZnO-nanoparticles on mycotoxins production

The lowest concentration of ZnO-NPs that showed strong inhibition (150 ppm) was chosen to assess its impact on mycotoxin production by toxigenic fungi. ZnO-NPs were added to 250 flasks containing YES broth for aflatoxin production and to modified AAL toxin medium at a final concentration of 150 ppm. The flasks were autoclaved at 121 °C for 20 min, and after autoclaving, the culture media were inoculated with a spore suspension (10^7^ CFU/mL) of the toxigenic strains, *A. flavus* and *F. proliferatum*. The inoculated flasks were then incubated at 28 ± 2 °C for 14 days for *A. flavus* and 21 days for *F*. *proliferatum*. After the incubation period, the contents of each flask were filtered, and the filtrates were used to measure mycotoxin levels using HPLC technique^58^.

### Statistical analysis

Statistical analyses were conducted using GraphPad Prism software version 5. Data are presented as means ± standard errors of the mean (SEM) and were normally distributed. A one-way ANOVA was performed to assess significant differences among the three groups, followed by Tukey’s post hoc test.

## Data Availability

All the generated data and the accession number for all the tested microorganisms are included in the manuscript.
